# Thrombin-derived C-terminal peptides bind and form aggregates with sulfated glycosaminoglycans

**DOI:** 10.1016/j.heliyon.2024.e35703

**Published:** 2024-08-02

**Authors:** Ganna Petruk, Jitka Petrlova, Firdaus Samsudin, Peter J. Bond, Artur Schmidtchen

**Affiliations:** a Division of Dermatology and Venereology, Department of Clinical Sciences, Lund University, 22241, Lund, Sweden; b Department of Biomedical Science, Faculty of Health and Society, Malmö University, SE-205 06, Malmö, Sweden; c Bioinformatics Institute (BII), Agency for Science, Technology and Research (A*STAR), 30 Biopolis Street, #07-01 Matrix, Singapore, 138671, Republic of Singapore; d Department of Biological Sciences, National University of Singapore, Singapore, 117543, Republic of Singapore; e Dermatology, Skane University Hospital, 22185, Lund, Sweden

## Abstract

Glycosaminoglycans (GAGs) such as heparin and heparan sulfate (HS) play crucial roles in inflammation and wound healing, serving as regulators of growth factors and pro-inflammatory mediators. In this study, we investigated the influence of heparin/HS on thrombin proteolysis and its interaction with the generated 11 kDa thrombin-derived C-terminal peptides (TCPs). Employing various biochemical and biophysical methods, we demonstrated that 11 kDa TCPs aggregate in the presence of GAGs, including heparin, heparan sulfate, and chondroitin sulfate-B. Circular dichroism analysis demonstrated that 11 kDa TCPs, in the presence of GAGs, adopt a β-sheet structure, a finding supported by thioflavin T1 (ThT) fluorescence measurements and visualization of 11 kDa TCP-heparin complexes using transmission electron microscopy (TEM). Furthermore, our investigations revealed a stronger binding affinity between 11 kDa TCPs and GAGs with higher sulfate group contents. Congruently, *in silico* simulations showed that interactions between 11 kDa TCPs and heparin/HS are predominantly electrostatic in nature. Collectively, our study suggests that 11 kDa TCPs have the capacity to aggregate in the presence of GAGs, shedding light on their potential roles in inflammation and wound healing.

## Graphical abstract

**Figure:**
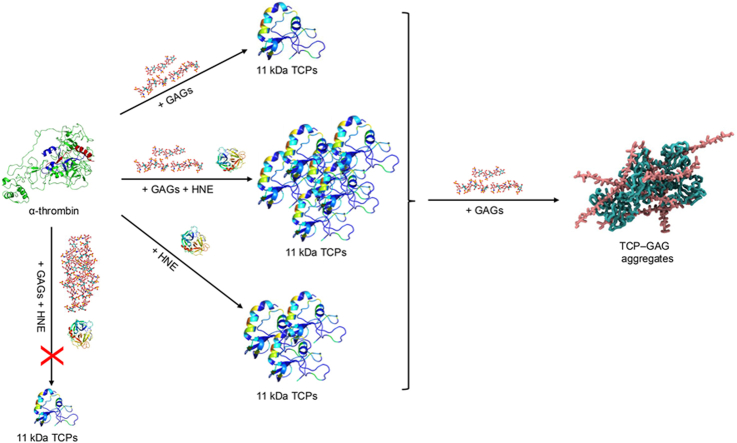

## 1 Introduction

Injured tissues contain damage-associated molecular patterns (DAMPs), which are regarded as endogenous danger signals that induce strong inflammatory responses [1,2]. Neutrophils, guided by DAMPs, are the first blood cell types that enter the area of injury [3]. These immune cells amplify the inflammatory response by releasing high amount of toxic effectors, including reactive oxygen species (ROS), various pro-inflammatory mediators, and proteases, including neutrophil elastase (NE) [4]. In the context of wound healing, the primary role of these molecules is to eradicate contaminating bacteria and facilitate tissue remodeling [5]. However, prolonged neutrophil activity at the injury site can also lead to tissue damage [6]. For example, NE, if uncontrolled, contributes to the excessive degradation of extracellular matrix components (ECM) like elastin and collagen, thereby promoting tissue damage.

In our prior investigations, we revealed that the proteolytic action of human NE (HNE) on thrombin results in the production of thrombin-derived C-terminal peptides (TCPs), which are approximately 2 kDa in size [7]. These TCPs have been identified in wound fluids and have demonstrated anti-endotoxic properties both *in vitro* and *in vivo*. In addition to these smaller fragments, the proteolysis of thrombin by HNE also gives rise to 11 kDa TCPs, found in wound environments [8]. Notably, our work unveiled the ability of these 11 kDa TCPs to aggregate in the presence of lipopolysaccharide (LPS) and *E. coli* bacteria. This aggregation leads to confinement of LPS and the elimination of bacteria *in vitro* as well as in experimental animal models [8,9].

Proteases can also release glycosaminoglycans (GAGs) such as heparan sulfate (HS) and chondroitin sulfate (CS) [10]. CS is composed of repeating disaccharide units of N-acetylgalactosamine (GalNAc) and glucuronic (GlcA) acid, with variable sulfation patterns primarily at the 4- and 6-positions of the GalNAc. CS-B, also called dermatan sulfate, contains the epimer of GlcA, iduronic acid (IdoA). HS is a complex GAG containing repeating disaccharide units of uronic acid (GlcA or IdoA) and glucosamine (GlcN), extensively modified by N-sulfation, O-sulfation, and N-acetylation. These modifications create diverse sulfation patterns, while epimerization of GlcA acid to IdoA acid adds further structural diversity. HS chains contain distinct sulfated and non-sulfated domains, contributing to functional heterogeneity and conformational flexibility [11,12].

HS exerts multifaceted roles in inflammation. It is expressed in the extracellular matrix and on various cell surfaces, including immune cells. HS plays a pivotal role in directly stimulating immune cells during inflammation. Enzymes can release soluble HS fragments that act as DAMPs, particularly through Toll-like receptor 4 (TLR-4), alerting the immune system to tissue damage, contributing to immune activation during inflammation [13]. Thus, *in vivo* studies have showed the importance of the HS-TLR-4 axis in experimental pancreatitis [14]. Additionally, HS participates in the *trans*-presentation of growth factors and inflammatory mediators to target cells, influencing signaling [15]. In summary, GAGs, have multifaceted roles in various biological processes. Their specific interactions with proteins are influenced by charge distribution from sulfate and carboxylate groups and the GAG chain conformation, ranging from nonspecific to highly specific based on unique structural features [11,16].

With this as background, we decided to investigate the possible interaction between TCPs and GAGs, such as heparin/HS and its potential implications for immune responses and inflammation *in vivo*. Understanding this interaction can provide insights into the potential modulatory effects of TCPs on HS function.

## 2 Results

### 2.1 TCPs in wound fluids and generation *in vitro*

**Fig. 1:**
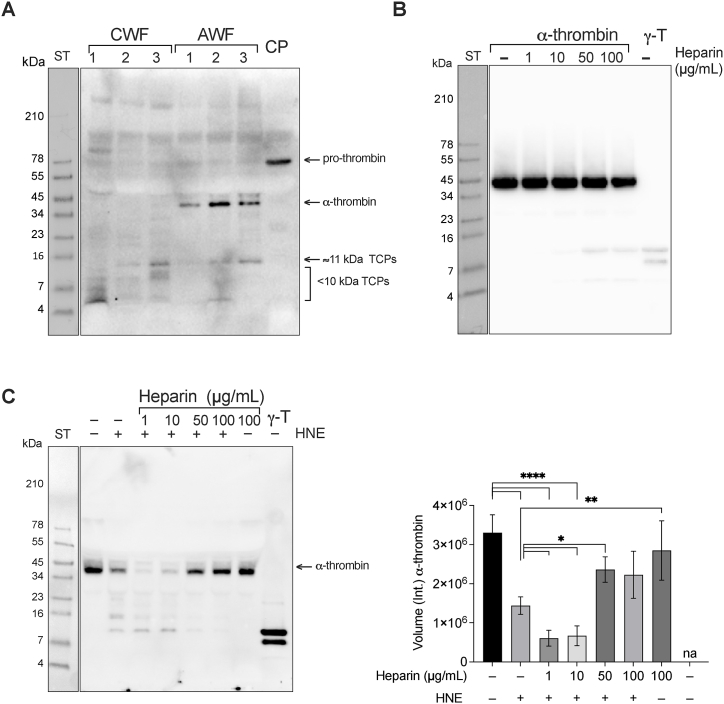
**11 kDa TCPs in wound fluid and generation *in vitro.*** (**A-C**) Representative image of SDS-PAGE, followed by western blotting using specific antibodies against the C-terminal thrombin epitope VFR17. (**A**) Three chronic wound fluids (CWF), from wounds colonized with 1, 2 or 3 bacterial strains, and three acute wound fluids (AWF), containing 0, 20 or 40 μg/mL GAGs, respectively. Human citrate plasma (CP) was used as a control. (**B**) Human α-thrombin alone or in combination with increasing concentrations (1–100 μg/mL) of heparin. γ-thrombin (γ-T) was used as a control. (**C**) Human serum incubated with human neutrophil elastase (HNE) alone or with increasing concentrations of heparin. Serum with only 100 μg/mL heparin and γ-thrombin (γ-T) were used as controls. The intensity of the bands at 42 kDa, corresponding to the molecular mass of α-thrombin, was quantified using Image Lab software 6.1 from Bio-Rad. Experiments in (**B**) and (**C**) were performed at least 3 times (n ≥ 3)*,* and * indicates *P <*0.05, ** indicates *P <*0.01, **** indicates *P <*0.0001. *P* values were determined using one-way ANOVA with Dunnett's multiple comparison test.

Chronic wound fluids (CWF), infected with 1, 2, or 3 bacterial strains, and acute wound fluids (AWF), containing 0, 20, or 40 μg/mL GAGs, were analyzed on SDS-PAGE, followed by Western blot. We observed fragments with a similar migration corresponding to a molecular mass of 11 kDa TCPs (i.e., γ-thrombin) in both WF types (Fig. 1A).

The observation that 11 kDa TCPs were detected in the sterile WFs containing GAGs, prompted us to investigate whether the presence of GAGs could induce thrombin fragmentation. We therefore incubated human α-thrombin with increasing doses of the highly sulfated GAG heparin (0–100 μg/mL) followed by Western blot analysis. As shown in Fig. 1B, α-thrombin was undergoing proteolysis in a dose dependent manner, suggesting a link between GAGs, proteolytic degradation, and formation of 11 kDa TCPs in wound fluids.

**Fig. 2:**
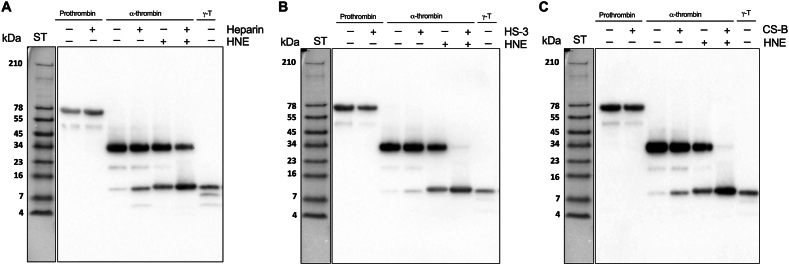
**Effects of GAGs on α-thrombin digestion by HNE.** Representative images of SDS-PAGE followed by Western blot show increased proteolysis of α-thrombin by HNE in the presence of heparin (**A**), heparan sulfate (HS–3) (**B**) and chondroitin sulfate B (CS–B) (**C**). Prothrombin and γ-thrombin (γ-T) were used as controls. All experiments were performed at least 3 times (n ≥ 3).

To explore heparin's potential to induce thrombin degradation under physiological conditions, we initiated thrombin generation by allowing human blood to coagulate. Subsequently, varying concentrations of heparin (ranging from 0 to 500 μg/mL) were added for further examination. After incubation at 37 °C, the soluble fraction was analyzed by SDS-PAGE followed by Western blot. We observed a dose-dependent decrease in the intensity of α-thrombin (Fig. S1A). However, we did not note any increase in fragments of 11 kDa TCPs. To explore whether α-thrombin is degraded in a cell-free environment, we next repeated the same experiment, but replaced blood with serum. The results showed that no degradation of α-thrombin was detected (Fig. S1B). During blood coagulation, neutrophils release elastase in amounts that can exceed 100 nmol/L [17], and moreover, neutrophil elastase is released in wounds and found in wound fluids. In order to mimic this physiological environment, we therefore explored the effects of GAGs and human neutrophil elastase (HNE) on α-thrombin. We incubated human serum with HNE alone, or with increasing doses of heparin. At lower doses of heparin, degradation of α-thrombin was increased, with almost complete disappearance of the intact enzyme after addition of 1 and 10 μg/mL heparin, while at higher doses no such degradation was observed (Fig. 1C). This is in line with previous studies, showing that heparin is known to inhibit activity of HNE [18,19]. To further study the combined effects of GAGs and HNE on thrombin, we next used a reductionist system, by incubating pure α-thrombin in the presence of heparin, a medium-sulfated HS form (HS-3), and CS-B, with and without HNE. Subsequently, we analyzed the samples by SDS-PAGE followed by Western blot. As shown in Fig. 2, incubation of thrombin with heparin (A), HS–3 (B), or CS–B (C), yielded a distinct band at around 11 kDa, corresponding to γ-thrombin. This fragmentation was not detected using prothrombin (Fig. 2) or in the presence of protease inhibitors (Fig. S2), indicative of a dependence of thrombin-mediated proteolysis. Incubation of α-thrombin with HNE yielded a similar 11 kDa fragment. However, when α-thrombin was digested by HNE in the presence of GAGs, an almost complete degradation of thrombin was detected accompanied by a concurrent increase in the intensity of the band at 11 kDa.

Taken together, these results demonstrate that 11 kDa TCPs are found in human wound fluids from acute and non-healing wounds. A dose-dependent GAG-induced proteolysis of α-thrombin, leading to the generation of 11 kDa TCPs, was observed using simple buffer conditions, and the addition of HNE further increased this degradation. No evident generation of 11 kDa TCPs was observed in blood or serum after the addition of heparin alone. However, mimicking wound fluid conditions by adding heparin in the presence of HNE demonstrated a boosted thrombin degradation and 11 kDa TCP generation, particularly at low heparin concentrations similar to physiological GAG levels, with marked inhibition in serum conditions using higher doses of heparin.

### 2.2 TCPs aggregate in the presence of heparin

GAGs, with the exception for hyaluronic acid, contain different sulfate substituents at various positions of the chain. Heparin, the most sulfated GAG [20], was used to investigate its effect on a recombinantly produced 11 kDa TCP [8] (referred to as rTCP_96_).

**Fig. 3:**
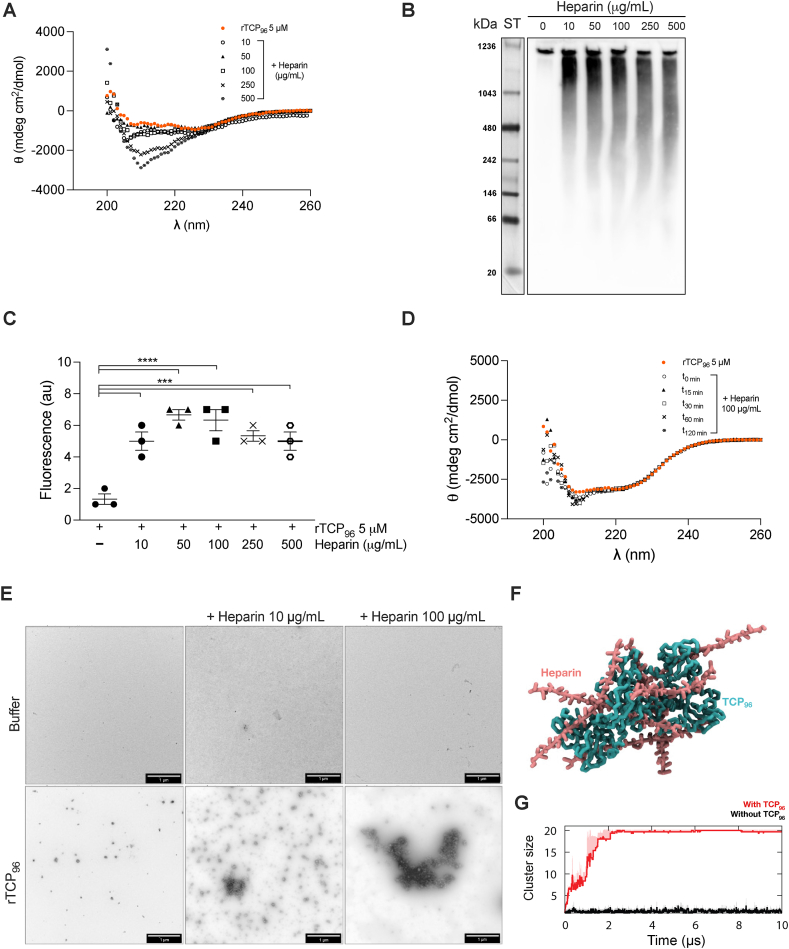
**Evaluation of binding of recombinantly produced 11 kDa TCPs (rTCP**_**96**_**) to heparin**. (**A** and **B**) Representative CD spectra showing a change in secondary structure of 5 μM rTCP_96_ with increasing concentrations (0–500 μg/mL) of heparin (**A**) or after different incubation times (**D**). (**B**) Representative BN-PAGE image followed by Western blot showing an increased migration of rTCP_96_ with higher doses of heparin. (**C**) Increasing fluorescence in the ThT assay indicates β-sheet formation in 5 μM rTCP_96_ in the presence of increasing concentrations of heparin. (**E**) TEM images showing an increase in the aggregated form of rTCP_96_ in the presence of heparin. Scale bar is 1 μm. All experiments were performed at least 3 times (n ≥ 3), and *** indicates *P* < 0.001, **** indicates *P* < 0.0001. *P* values were determined using one-way ANOVA with Dunnett's multiple comparison test. **(F)** The final snapshot from a CG simulation of 10 heparin (pink) with 10 TCP_96_ (cyan). **(G)** Cluster size indicating the number of molecules forming the largest single cluster throughout three independent 10 μs simulations using a distance cut-off of 0.6 nm (red), compared to simulations of heparin without TCP96 (black). Average values are shown as thick lines and standard deviations between repeats are shown as shaded areas.

First, we used circular dichroism (CD) to study possible changes in the secondary structure of the peptide (5 μM) in presence of varying concentrations of heparin (0–500 μg/mL). Fig. 3A displays representative CD spectra, illustrating a dose-dependent transition in the secondary structure of rTCP_96_. In its standalone state, rTCP_96_ exhibited a typical α-helical spectrum (red dots). However, in the presence of heparin, a discernible change in secondary structure was observed. The binding of rTCP_96_ to heparin was further confirmed by BN–PAGE (Fig. 3B). Considering the theoretical pI of the peptide (7.2) and the neutral pH of the electrophoresis conditions, no migration in the gel of the peptide alone was expected. However, with increasing heparin doses, a pronounced shift in protein migration towards lower molecular weights was observed, indicating the introduction of a highly negative charge to the peptide. Thioflavin T1 (ThT) fluorescence probe was employed to monitor the ability of rTCP_96_ to adopt a β-sheet structure, typical of amyloids, upon incubation with heparin. Fig. 3C displays the fluorescence intensity at 482 nm for the peptide alone and in conjunction with heparin. Additionally, CD analysis was utilized to examine how the incubation time of rTCP_96_ with heparin affected its secondary structure. Fig. 3D shows that the change is almost imperceptible. Finally, the aggregation of rTCP_96_ with heparin was visualized by transmission electron microscopy (TEM) (Fig. 3E). Notably, the aggregates of the peptide were more prominent with 100 μg/mL of heparin compared to 10 μg/mL. To understand the molecular basis of TCP_96_ aggregation with heparin, we performed coarse-grained (CG) molecular dynamics (MD) simulations of ten copies of 20-mer heparin strands with ten copies of TCP_96_ (Fig. 3F). Indeed, we observed all heparin strands and TCP_96_ peptides forming a single cluster within the first 3 μs of the simulations (Fig. 3G). In contrast, simulations without TCP_96_ peptides showed no aggregation among the heparin strands, in agreement with our TEM results (Fig. 3E). Contact analysis revealed that helix 80–96 of TCP_96_ peptide made significant interactions with heparin throughout the simulations (Fig. S3), congruent with the putative heparin binding site on thrombin [21].

### 2.3 Binding and aggregation of 11 kDa TCPs in the presence of different GAGs

**Fig. 4:**
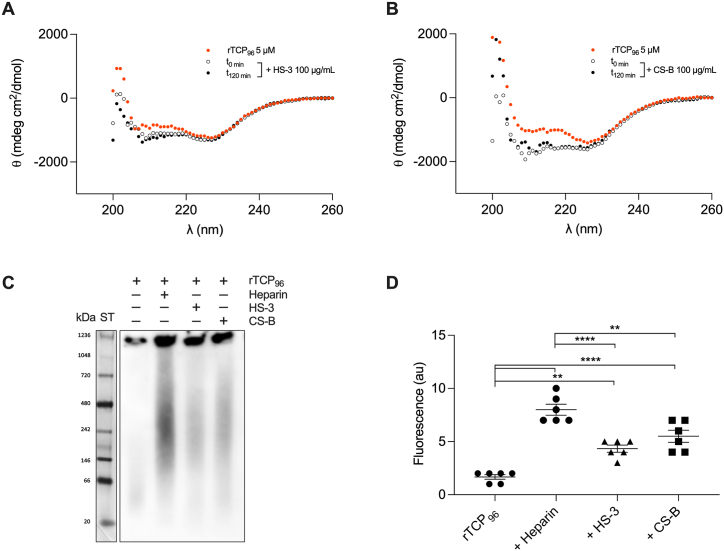
**Evaluation of binding of rTCP**_**96**_**to GAGs**. (**A** and **B**) Representative CD spectra for 5 μM rTCP_96_ in the presence of 100 μg/mL of heparan sulfate (HS–3) and chondroitin sulfate B (CS–B), immediately or after 120 min of incubation. (**C**) Representative image of BN-PAGE followed by Western blot showing an increase in the migration of rTCP_96_ in the presence of different GAGs. (**D**) ThT assay of 5 μM rTCP_96_ in the presence of 100 μg/mL of heparin, HS–3 or CS–B. All experiments were performed at least 3 times (n ≥ 3), and ** indicates *P* < 0.005, **** indicates *P* < 0.0001. *P* values were determined using one-way ANOVA with Tukey's multiple comparison test.

Following the results obtained with heparin, we decided to assess the binding capacity of rTCP_96_ to other GAGs, specifically HS–3 and CS–B. The secondary structure of 5 μM rTCP_96_ was influenced by both GAGs under test (Fig. 4A and **B**). Similar to the results with heparin, the conformational change in the secondary structure of the peptide did not depend on the incubation time. The binding affinity of rTCP_96_ to GAGs was confirmed by BN-PAGE (Fig. 4D). Indeed, a more pronounced shift in migration of rTCP_96_ was observed for the peptide incubated with heparin. The aggregation of rTCP_96_ into amyloids in the presence of HS–3 and CS–B was validated by ThT assay (Fig. 4C).

**Fig. 5:**
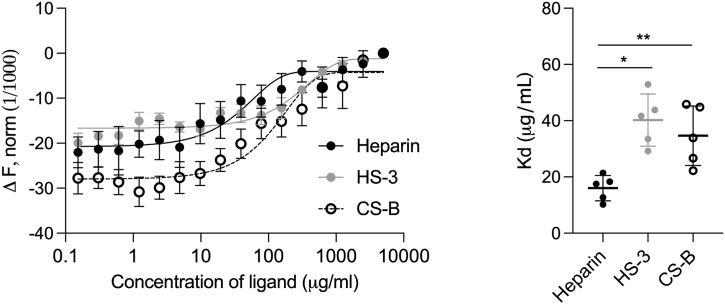
**Evaluation of binding affinity of rTCP**_**96**_**to different GAGs**. (**Left**) Representative microscale thermophoresis (MST) curves of rTCP_96_ with increasing concentrations of heparin, heparan sulfate (HS–3) and chondroitin sulfate B (CS–B). (**Right**) Kd values were obtained from MST curves. Kd of 16.0 ± 4.5, 40.2 ± 9.3 and 34.7 ± 10.6 μg/mL were determined for heparin, HS–3 and CS–B, respectively. Mean ± SD values of five measurements are shown (n = 5). *P* values were determined using one-way ANOVA with Tukey's multiple comparison test. * indicates *P* < 0.05, ** indicates *P* < 0.005.

To study the binding affinity, we incubated the labeled peptide in the presence of increasing concentrations of different GAGs and performed microscale thermophoresis (MST). In Fig. 5, binding curves (left) and Kd values (right) for heparin, HS–3 and CS–B are reported. In line with previous results, the binding affinity of heparin to rTCP_96_ is stronger than observed for both HS–3 and CS–B.

### 2.4 Influence of sulfation ratio of GAGs on binding affinity to 11 kDa TCPs

Previously, we have shown that *E. coli* lipopolysaccharide (LPS) predominantly binds to the C-terminal region of thrombin, mainly through interaction with positively charged amino acid residues [22,23]. Building on this, we hypothesized that a similar interaction could apply to GAGs. To investigate this, we used HS with varying degrees of sulfate groups [24].

**Fig. 6:**
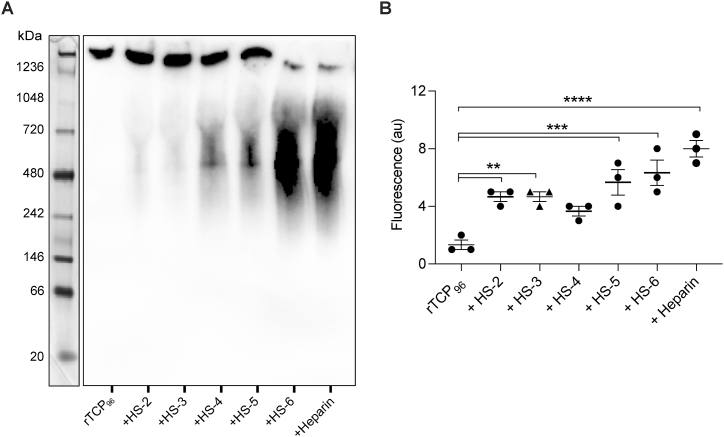
**Evaluation of binding capacity of highly sulfated GAGs to 11 kDa TCPs**. (**A**) Representative image of BN-PAGE followed by Western blot showing an increase in migration of rTCP_96_ in the presence of highly sulfated GAGs. (**B**) Increasing fluorescence in ThT assay indicates β-sheet formation in 5 μM rTCP_96_ in the presence of GAGs with increasing sulfate group ratio. All experiments were performed at least 3 times (n ≥ 3) *P* values were determined using one-way ANOVA with Dunnet's multiple comparison test*.* ** indicates *P* < 0.00*5,* *** indicates *P <*0.001, and **** indicates *P* < 0.0001.

We first confirmed the binding by BN-PAGE followed by Western blot (Fig. 6A). As anticipated, GAGs with a higher number of sulfate groups induced an increase in peptide migration. Subsequently, we validated the aggregation of rTCP_96_ in the presence of these GAGs using the ThT assay (Fig. 6B).

**Fig. 7:**
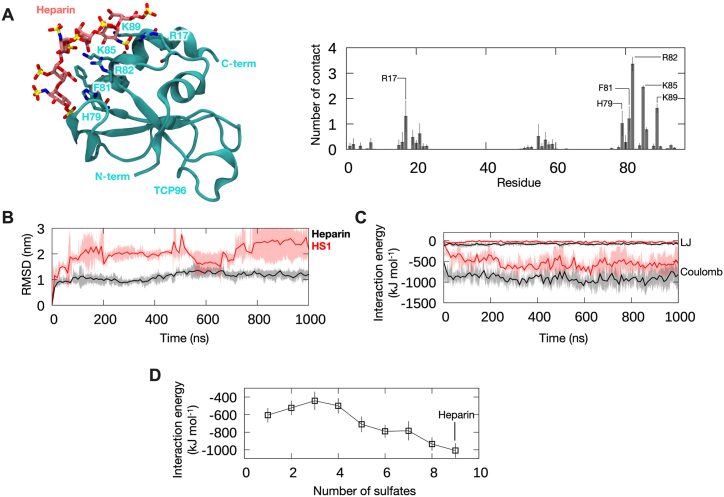
**Molecular dynamics simulation of 11 kDa TCP with heparin and HS.** (**A**) (**Left**) A representative structure of 11 kDa TCP (TCP_96_) -heparin complex. A clustering analysis was performed on concatenated trajectories of three independent 1000 ns simulations using an RMSD cut-off of 0.4 nm, and the central structure of the top cluster is shown. Peptide is shown in cartoon representation in cyan and heparin is shown in stick representation in pink. Peptide residues that made most interactions with heparin are labeled. (**Right**) Average number of contacts made by each peptide residue with heparin from the simulations. Error bars show standard deviations between repeats. Residues making significant contact are labeled. Cut-off distance for contact analysis is 0.4 nm. (**B**) Average root means square deviation (RMSD) of heparin (black) and HS with one sulfate group (HS–1, red) after least square fitting to the TCP_96_-heparin/HS1 complex. Shaded areas indicate standard deviations between repeats. (**C**) Average Lennard-Jones (LJ) and Coulombic interaction energies between TCP_96_ and heparin or HS–1 throughout the simulations. (**D**) Average sum of interaction energies (LJ plus Coulombic components) calculated from the last 500 ns of the simulations, comparing between heparin with 9 sulfate groups and HS with 1–8 sulfate groups. Error bars show standard deviations between three repeat simulations.

These findings were further supported by *in silico* modeling and all-atom MD simulations of TCP_96_ bound to heparin (Fig. 7A). Our simulations showed that the interactions between the peptide and heparin are predominantly electrostatics, with Lys/Arg residues making most contacts to the negatively charged sulfate groups (Fig. 7A). To model the binding of TCP_96_ to HS with different numbers of sulfate groups, we then gradually removed the sulfate groups from heparin molecule in the initial TCP_96_-heparin complex (details in STAR Methods). RMSDs were measured to determine the stability of binding, and interaction energies were calculated as a proxy for binding affinities. Comparing heparin to HS with 1 sulfate group, we observed that heparin binds more stably, as demonstrated by the lower RMSD, and with higher affinity, as indicated by the more negative interaction energies with the peptide (Fig. 7B and **C**). When the sulfate groups were gradually removed from heparin, we observed a decreasing interaction energy with TCP_96_ (Fig. 7D), indicating that the more sulfate groups the heparin or HS contains, the higher the binding affinities to the TCP_96_, in agreement with experimental results in Fig. 6.

## 3 Discussion

Even the slightest skin injury can inflict significant harm on the host. Consequently, evolution has endowed the host with multiple intersecting defense mechanisms. One of these has thrombin as a key player. Indeed, thrombin has a pivotal role in fibrinogen degradation and clot formation, in hemostasis and other physiological functions in innate defense [25]. Proteolysis of thrombin generates TCPs, which occur naturally in human wounds [[26], [27], [28], [29]]. The length of these peptides is associated with different functions, smaller 2–3 kDa peptides exert antimicrobial and anti-inflammatory effects, whereas larger fragments such as the ones described in this report mediate aggregation [8,9,[26], [27], [28], [29]]. From an evolutionary perspective, this is an efficient way of the host to re-use parts of an enzyme once it has fulfilled its primary function, i.e., the first line of defense. Previously, it was demonstrated that 11 kDa TCPs were able to aggregate bacteria and their endotoxins, facilitating the clearance through phagocytosis [8,9]. Thus, the observation that 11 kDa TCPs were detected in various wound fluids (Fig. 1A) was not surprising and aligns with the proposed physiological role for such peptides.

Several studies have shown that heparin and other GAGs can enhance protease action by sequestering their inhibitors [6], as well as inhibit their action through high-affinity binding [30,31]. Here, we present evidence that GAGs can induce proteolysis of thrombin (Fig. 1, Fig. 2), generating 11 kDa TCPs. However, this phenomenon was only observed in simple buffer conditions, and not in the presence of a blood or serum environment. Since the two enzymes α-thrombin and HNE, along with variable levels of GAGs, are all present in wounds, the interplay between these factors may determine the overall kinetics of thrombin proteolysis, and hence, generation of different TCPs *in vivo*. It is therefore notable that at physiological levels of HNE (2.89 ± 1.29 U/mL) [32,33] and heparin (1–24 μg/mL) [34], the proteolysis of α-thrombin increases in human serum, with concomitant release of 11 kDa TCPs (Fig. 1C), which is well in line with such a mechanism.

The observation that thrombin fragmentation is inhibited at higher concentration of GAGs (Fig. 1C), is also compatible with previous studies on modulation of HNE and thrombin activity by heparin and other GAGs [30,31,35]. Taken together, these data illustrate that the roles of heparin and other GAGs in proteolysis control are both context and concentration dependent.

Using several biochemical and biophysical methods as well as *in silico* CG simulations, we have shown that 11 kDa TCPs aggregate in the presence of GAGs (Fig. 3, Fig. 4). This suggests that 11 kDa TCPs may play a role in confinement and clearance of GAGs, analogously to its function in scavenging endotoxins, curbing excessive inflammation [8,9]. The binding between GAGs and 11 kDa TCPs has an electrostatic component, as highly sulfated GAGs bound to the peptide with higher affinity (Fig. 5, Fig. 6, Fig. 7). This aligns with previous studies showing that highly anionic linear polysaccharides can interfere with the activity of cationic proteins, such as NE, *via* charge interactions [31,36]. For example, heparin and its derivatives can inhibit elastase activity in the range of 40 nM−100 μM, based on the chain length of the oligosaccharides [37]. In this study, we found that the binding affinity between 11 kDa TCPs and heparin, HS-3 and CS-B was 16.0 ± 4.5, 40.2 ± 9.3 and 34.7 ± 10.6 μg/mL, respectively, corresponding to around 1, 1.4 and 1.8 μM (considering the average MW to be 15, 30 and 20 kDa, respectively). Interestingly, observations have demonstrated that GAGs such as CS-B, released in wounds, reach concentrations in the range of 15–65 μg/mL [38], indicating that under physiological conditions, GAGs can modulate and affect TCP generation as well as aggregation.

In conclusion, our research reveals that 11 kDa TCPs not only aggregate in the presence of bacteria and their endotoxins but also exhibit the capability to aggregate when combined with GAGs. Furthermore, our findings emphasize the complex modulatory actions of GAGs on generation of 11 kDa TCPs, underscoring an intricate interplay between HNE, thrombin, and GAGs. This relationship holds significance in the context of immune responses and the regulation of inflammation.

## 4 Limitations of the study

While this study demonstrates that GAGs can induce proteolysis of thrombin and aggregation of 11 kDa TCPs, it does not elucidate the underlying molecular mechanisms governing these processes. Additional research is required to comprehensively understand these mechanisms. Furthermore, the levels of HNE and GAGs in fluids derived from chronic infected wounds, used in this study, were not analyzed. Moreover, whether aggregation of 11 kDa TCPs in the presence of GAGs might play a role in GAG clearance needs further *in vitro* validation, followed by an exploration of its broader implications *in vivo.* Lastly, to study specific structural requirements for TCP interaction, chemically modified heparin preparations could be used in interaction studies. For instance, using N-desulfated heparin, 2-O-desulfated heparin, and 6-O-desulfated heparin preparations could provide insights into the specificities of the interaction with TCPs. Additionally, defining the sequences and bioactive motifs in GAGs that interact with TCPs could be achieved through combinations of affinity chromatography and various GAG sequencing approaches [[39], [40], [41]].

## Star methods

Detailed methods are provided in the online version of this paper and include the following:

• KEY RESOURCE TABLE;
• RESOURCE AVAILABILITY;
  o Lead contact
  o Materials availability
  o Data and code availability
• EXPERIMENTAL MODEL AND STUDY PARTICIPANT DETAILS;
  o Human participants
  o Ethics statement
• METHOD DETAILS;
  o Proteins and peptides
  o Heparan sulfate preparation
  o Electrophoresis and western blot
  o Thioflavin T1 (ThT) assay
  o Circular Dichroism Spectroscopy
  o Transmission Electron Microscopy
  o Coarse-grained molecular dynamics simulation of TCP_96_ aggregation with heparin
  o All-atom molecular dynamics simulation of TCP_96_ with heparin and heparan sulfate
  o Microscale thermophoresis (MST)
• QUANTIFICATION AND STATISTICAL ANALYSIS.

## Inclusion and diversity

We support inclusive, diverse and equilibrate conduct of research.

## Star methods

**Table:** Key resources table

REAGENT or RESOURCE	SOURCE	IDENTIFIER
Antibodies		
Polyclonal rabbit antibodies against the VFR17 epitope	Innovagen AB	http://www.innovagen.com/products
Anti-rabbit HRP-conjugated antibodies	Dako	P0217
Bacterial and virus strains		
*Escherichia coli* strain BL21 codonplus (DE3) RIPL	Invitrogen	C600003
Biological samples		
Healthy donors	Lund University	https://www.staff.lu.se/research-and-education/research-support/research-ethics-and-animal-testing-ethics
Sterile acute wound fluids	Lund University	https://www.staff.lu.se/research-and-education/research-support/research-ethics-and-animal-testing-ethics
HS-2, HS-3, HS-4, HS-5, HS-6	HS fractions were obtained from Dr L-Å Fransson. The preparation of these has been described previously (Fransson L.-Å, Sjöberg, I. and Havsmark, B. (1980), Structural Studies on Heparan Sulphates. European Journal of Biochemistry, 106: 59–70. https://doi.org/10.1111/j.1432-1033.1980.tb05997.x)	
Chemicals, peptides, and recombinant proteins		
Chloramphenicol	Duchefa Biochemie	C01130025
Carbomycin	Smolecules	S522695
IPTG	Swab	I1000-5
Tris	AppliChem	A1086,5000
LB medium	Sigma-Aldrich	L3022-1 KG
Urea	Duchefa Biochemie	U1363.5000
Imidazole	VWR Life Science	0527-100G
The thrombin-derived peptide TCP-25	AmbioPharm, Inc.	https://www.ambiopharm.com
Human α-thrombin	Innovative RESEARCH	https://www.innov-research.com
Human prothrombin	Innovative RESEARCH	https://www.innov-research.com
Human γ-thrombin	Innovative RESEARCH	https://www.innov-research.com
Human neutrophil elastase	Sigma-Aldrich	324681
Heparin	Sigma-Aldrich	H3393–100KU
Chondroitin sulfate B	Sigma-Aldrich	C-3788
10–20 % Novex Tricine pre-cast gel	Life Technologies	EC6625BOX
NativePAGE Bis-Tris Gels System 4–16 %	Life Technologies	BN2111BX10
*Trans*-Blot® Turbo™ Mini PVDF Transfer Packs	Bio-Rad	170–4156
SuperSignal West Pico Chemiluminescent Substrate	Thermo Scientific	34080
Thiflavin T	Sigma-Aldrich	T3516-5G
Software and algorithms		
Image Lab 6.1	Bio-Rad Laboratories, Inc.	https://www.bio-rad.com/en-se/product/image-lab-software?ID=KRE6P5E8Z&s_kwcid=AL%2118120%213%21657783331339%21e%21%21g%21%21image+lab+6.1&WT_mc_id=240108040415&WT_srch=1&WT_knsh_id=_kenshoo_clickid_&gad_source=1&gclid=CjwKCAiAlcyuBhBnEiwAOGZ2Sx1wEDhwRKZie6RbuYzgJKZn3w8TNxw_5KpD6eLJtSmUDWRTTEcZkxoCRhQQAvD_BwE
GraphPad Prism v10.0.	GraphPad Software	https://www.graphpad.com/features
Spectra Manager™ Suite Spectroscopy Software	Jasco Inc.	https://jascoinc.com/products/spectroscopy/molecular-spectroscopy-software/
MO.Affinity Analysis Software	NanoTemper	https://shop.nanotempertech.com/en/moaffinity-analysis-software-unlimited-licenses-34
Modeller v9.21	Sali and Blundell (1994)	https://salilab.org/modeller/
CHARMM-GUI Solution Builder	Jo et al. (2008)	https://charmm-gui.org/?doc=input/solution
CHARMM-GUI Glycan Reader and Modeller	Park et al. (2019)	https://charmm-gui.org/?doc=input/glycan
GROMACS 2021	Abraham et al. (2015)	https://www.gromacs.org
VMD 1.9	Humphrey and Dalke (1996)	https://www.ks.uiuc.edu/Research/vmd/
Other		
Carbon-coated grids (copper mesh, 400)	TED PELLA, INC.	01754–7
Immobilized Metal Affinity Chromatography (IMAC) (His-Trap-Nickel-chelating columns	Invitrogen	R901-15

## Resource availability

### Lead contact

Further information and requests for materials should be directed to and will be fulfilled by the lead contact, Ganna Petruk (ganna.petruk@med.lu.se)

### Materials availability

This study did not generate new unique reagents.

### Data and code availability

This paper does not report original code.

Any additional information required to reanalyze the data reported in this paper is available from the lead contact upon request.

## Experimental model and study participant details

### Human participants

Chronic wound fluids (CWF) were collected from patients with non-healing venous ulcers colonized with only *Pseudomonas aeruginosa* (*P. aeruginosa*), with *Staphylococcus aureus* (*S. aureus*) and *P. aeruginosa*, or with Group A *β-streptococcus*, *P. aeruginosa* and *S. aureus*, as described previously [29]. Acute wound fluids (AWF), with 0, 20 and 40 μg/mL GAGs, were obtained from surgical drainages after surgery [8]. All wound fluids were centrifuged, aliquoted, and stored at −20 °C. Human blood was collected in from healthy volunteers. After collection, whole blood or its fraction, such as plasma and serum, were used immediately or stored at −80 °C.

### Ethics statement

The use of human wound materials was approved by the Ethics Committee at Lund University (LU 708-01 and LU 509-01). The use of human blood was approved by the Ethics Committee at Lund University (permit no. 657–2008). Informed consent was obtained from all the donors.

## Method details

### Proteins and peptides

Human pro-, α- and γ-thrombin were purchased from Innovative Research, Inc. (Novi, MI, USA). Recombinant thrombin-derived C-terminal peptides (herein denoted rTCP_96_) were expressed and purified as reported by Petrlova et al. [8]. Briefly, pET-15b plasmid was inserted in *Escherichia coli (E. coli*) strain BL21 codonplus (DE3) RIPL (Invitrogen). Bacteria were cultivated in Luria Bertani (LB, Sigma_Aldrich) medium supplemented with antibiotics: chloramphenicol (34 μg/mL, Duchefa Biochemie) and carbomycin (100 μg/mL, Smolecules), at 37 °C with vigorous shaking. When the optical density at 620 nm (OD_620_) reached a value of 0.6 the expression of rTCP_96_ was induced by adding 0.25 mM Isopropyl β-d-1-thiogalactopyranoside (IPTG, Swab). After 3 h of incubation at 37 °C with vigorous shaking, bacteria were harvested by centrifugation (4000 g for 10 min), washed with 10 mM Tris at pH 7.4. The pellet derived from 0.5 L of bacterial culture was resuspended in 10 mL of 10 mM Tris at pH 7.4 containing 8 M urea and 20 mM imidazole (lysis buffer). Afterwards, the cells were disrupted by sonication (10 s ON and 10 s OFF, 10 times) and the soluble fraction, containing the peptides of interest, was separated from the cell debris by centrifugation (4000*g* for 20 min). Finally, the supernatant was filtered with a 0.22 μm syringe filter and diluted with other 10 mL of lysis buffer. rTCP_96_ was purified by Immobilized Metal Affinity Chromatography (IMAC) (His-Trap-Nickel-chelating columns, Invitrogen) under denaturing conditions (8 M urea in 10 mM Tris at pH 7.4). Eluted fractions containing rTCP_96_ were reunited, diluted with lysis buffer 1:1 and dialyzed stepwise in 10 mM Tris at pH 7.4 at 4 °C.

### Heparan sulfate preparation

HS fractions were obtained from Dr L-Å Fransson. The preparation and characteristics of these has been described previously [24]. Briefly, for HS-1 to HS-6, the total sulfate content ranged from 0.44 to 1.63 mol/mol hexosamine, with each fraction's total sulfate content at least twice that of N-sulfate. Fractions HS-1 to HS-4 had uronic acid to hexosamine ratios close to 1.00, while HS-5 and HS-6 had ratios above 1, similar to heparin. HS-1 to HS-4 contained more GlcUA than IdoUA, HS-5 had equal proportions, and HS-6 was IdoUA-rich like heparin. N-sulfate to hexosamine ratios were 0.21, 0.26, and 0.40 mol/mol for HS-1, HS-2, and HS-3, respectively, indicating minimal GlcUA-GlcNSO_3_ repeats. HS-5 and HS-6 had higher N-sulfate content, suggesting some GlcUA-GlcNSO repeats. The proportion of IdoUA increased with charge density. Finally, anticoagulant activity was negligible in HS-1 to HS-4, appreciable in HS-5, and similar to heparin in HS-6.

### Electrophoresis and Western blot

Chronic and acute wound fluids (100 μg total proteins) were mixed with loading buffer (Life technologies, Bleiswijk, NL), denatured at 95 °C for 10 min and then loaded on a 10–20 % Novex Tricine pre-cast gel (Invitrogen, Carlsbad, CA, USA). Electrophoresis was performed at 120 V for 90 min. Then, the material was transferred to a PVDF membrane using a *Trans*-Blot Turbo system (Bio-Rad, Laboratories, Hercules, CA, USA). Thrombin was detected using polyclonal rabbit antibodies against the C-terminal thrombin epitope VFR17 (VFRLKKWIQKVIDQFGE; diluted 1:1000, Innovagen AB, Lund, Sweden), followed by porcine anti-rabbit HRP conjugated antibodies (1:1000, Dako, Glostrup, Denmark). The peptide was visualized by incubating the membrane with SuperSignal West Pico Chemiluminescent Substrate (Thermo Scientific, Rockford, IL, USA) for 5 min, followed by detection using a ChemiDoc XRS Imager (Bio-Rad Laboratories, Hercules, CA, USA). Human citrated plasma (100 μg of total proteins) was used as a control.

Human α-thrombin (10 μM) was mixed with heparin (0–1–10–50–100 μg/mL, Sigma-Aldrich, Saint Louis, MO, USA) in 30 μL as final volume, and incubated for 30 min at 37 °C. At the end of incubation, samples were mixed with loading buffer (Invitrogen, Carlsbad, CA, USA) and 3 μL of 2-mercaptoethanol, denatured at 95 °C for 10 min and then 10 μL of each sample were loaded on a 10–20 % Novex Tricine pre-cast gel (Invitrogen, Carlsbad, CA, USA). Electrophoresis and Western blot were performed as described above. Human γ-thrombin was used as a control.

Human prothrombin or α-thrombin (10 μM) was mixed with 100 μg/mL of heparan sulfate (HS–3) or chondroitin sulfate B (CS–B, Sigma-Aldrich, Saint Louis, MO, USA) and then digested with human neutrophil elastase (HNE, Sigma-Aldrich, Saint Louis, MO, USA) for 30 min at 37 °C (30 μL as final volume). At the end of incubation, samples were mixed with loading buffer (Invitrogen, Carlsbad, CA, USA) and 3 μL of 2-mercaptoethanol, denatured at 95 °C for 10 min and then 10 μL of each sample were loaded on a 10–20 % Novex Tricine pre-cast gel (Invitrogen, Carlsbad, CA, USA). Electrophoresis and Western blot were performed as described above. Human γ-thrombin was used as a control.

Twenty microliters of rTCP_96_ (5 μM) were mixed with 10 μL of different concentrations of heparin (0–10–50–100–250–500 μg/mL) and 3 μL of 2-mercaptoethanol. Samples were incubated for 30 min at 37 °C, then 15 μL of each sample were loaded under reducing conditions on BN-PAGE (NativePAGE Bis-Tris Gels System 4–16 %, Invitrogen, Carlsbad, CA, USA). Electrophoresis was performed at 150 V for 100 min. Western blot was performed as described above. A Western blot of rTCP96 with different doses of heparin, or heparin alone, is shown in Fig. S4.

Twenty microliters of rTCP_96_ (5 μM) were mixed with 10 μL of different GAGs (HS-2,-3,-4,-5, -6, heparin, CS-B) (100 μg/mL) and 3 μL of 2-mercaptoethanol. Samples were incubated for 30 min at 37 °C, then 15 μL of each sample were loaded under reducing conditions on BN-PAGE (NativePAGE Bis-Tris Gels System 4–16 %, Invitrogen, Carlsbad, CA, USA). Electrophoresis and Western blot were performed as described above.

For experiments using human blood or serum, three different experimental setups were used. In the first, human blood (500 μL) was allowed to coagulate for 30 min at 37 °C, then heparin at increasing concentrations (0–1–10–20–50–100–500 μg/mL) was added. The samples were vortexed and incubated for 30 min at 37 °C. After incubation, the samples were centrifuged at 800*g* for 5 min. The serum was collected, and 2 μL of each sample were loaded under reducing conditions on SDS-PAGE (10–20 % Novex Tricine pre-cast gel, Invitrogen, Carlsbad, CA, USA). Electrophoresis and Western blot were performed as described above.

In the second setup, human blood was allowed to coagulate for 30 min at 37 °C, followed by centrifugation at 800*g* for 5 min. Two μL of collected serum were incubated with increasing concentrations of heparin (0–1–10–50–100 μg/mL) in a final volume of 20 μL. The samples were vortexed and incubated for 30 min at 37 °C. After incubation, the samples were loaded under reducing conditions on SDS-PAGE (10–20 % Novex Tricine pre-cast gel, Invitrogen, Carlsbad, CA, USA). Electrophoresis and Western blot were performed as described above. Human γ-thrombin was used as a control.

In the third setup, human blood was allowed to coagulate for 30 min at 37 °C, then it was centrifuged at 800*g* for 5 min. Two μL of collected serum were incubated with 2 μL of 0.1 μg/mL HNE alone, or in the presence of increasing concentrations of heparin (0–1–10–50–100 μg/mL) in a final volume of 20 μL. Samples were vortexed and incubated for 30 min at 37 °C. At the end of the incubation, the samples were loaded under reducing conditions on SDS-PAGE (10–20 % Novex Tricine pre-cast gel, Invitrogen, Carlsbad, CA, USA). Electrophoresis and Western blot were performed as described above. Human γ-thrombin and 2 μL of plasma with 100 μg/mL heparin were used as a control.

### Thioflavin T1 (ThT) assay

Aggregation of rTCP_96_ in the presence of heparin was evaluated by ThT assay. Five micromolar rTCP_96_ in 10 mM Tris at pH 7.4, alone or in combination with heparin (10–50–100–250–500 μg/mL) was incubated for 30 min at 37 °C. Then, 200 μL of each sample were incubated with ThT (final concentration of 10 μM) for 15 min in the dark. ThT fluorescence was measured using a VICTOR3 Multilabel Plate Counter spectrofluorometer (PerkinElmer) at an excitation of 450 nm, with 10 nm excitation and emission slit widths. Since ThT is known to bind to GAGs [42], the baseline (10 mM Tris buffer alone or in the presence of different concentrations of heparin) was subtracted from the signal of each sample to correct for this phenomenon [43].

Five micromolar rTCP_96_ in 10 mM Tris at pH 7.4, alone or in combination with 100 μg/mL of different GAGs was incubated for 30 min at 37 °C. Then ThT assay was performed as described above.

### Circular Dichroism Spectroscopy

The secondary structure of rTCP_96_ (5 μM) in 10 mM Tris at pH 7.4, alone or after addition of different concentrations of heparin (10–50–100–250–500 μg/mL) was determined by circular dichroism (CD). The measurements were performed on a Jasco J-810 spectropolarimeter (Jasco, Tokyo, Japan) equipped with a Jasco CDF-426S Peltier set to 25 °C. One centimeter quartz cuvette (Hellma GmbH & Co. KG, Müllheim, Germany) was used. The spectra were recorded at 200–260 nm (scan speed: 20 nm/min) as an average of 5 measurements. Raw spectra were corrected for buffer contribution and converted to the mean residue ellipticity, θ (mdeg cm^2^/dmol). The α-helical content was estimated according to the equation reported by Morrissette et al. [44].

To analyze the time dependence of the aggregation, rTCP_96_ (5 μM) in 10 mM Tris at pH 7.4 was incubated with 100 μg/mL heparin for different length of time (0–120 min) at 37 °C then spectra were recorded as described above.

To evaluate how HS-3 and CS-B affect the secondary structure of the peptide, the spectra of rTCP_96_ (5 μM) in 10 mM Tris at pH 7.4 alone, immediately after addition of 100 μg/mL of GAGs, or after 120 min of incubation at 37 °C, were recorded as described above.

### Transmission Electron Microscopy

The aggregates of rTCP_96_ in 10 mM Tris at pH 7.4, alone or with heparin were visualized by transmission electron microscopy (TEM) (Jeol Jem 1230; Jeol Ltd., Tokyo, Japan) in combination with negative staining. Briefly, 5 μM rTCP_96_ were incubated for 30 min at 37 °C with 10 and 100 μg/mL heparin. At the end of incubation, 5 μL of each sample were adsorbed onto carbon-coated grids (Copper mesh, 400) for 60 s and stained with 7 μL of 2 % uranyl acetate for 30 s [8]. The grids were rendered hydrophilic *via* glow discharge at low air pressure.

### Coarse-grained molecular dynamics simulation of TCP_96_ aggregation with heparin

The atomic structure of TCP_96_ was modelled based on the crystal structures of human α-thrombin (more details below). The atomic structure was converted to CG representation using the Martini 2.2 [45] and Martini 3 [46] forcefields with an elastic network applied to maintain the secondary and tertiary structures. The TCP_96_ CG models were first simulated alonein a box containing Martini water particles and 0.15 M NaCl salt. Energy minimization was performed using the steepest descent method followed by a 100 ns equilibration simulation whereby positional restraints with a force constant of 1000 kJ mol^−1^ nm^−2^ were applied to the backbone beads of the peptide. Three independent 1000 ns production simulations without restraints were performed for each forcefield. The temperature was maintained at 310 K using the velocity-rescaling thermostat [47], while the pressure was maintained at 1 atm using the Parrinello-Rahman barostat [48]. Coulombic interactions were calculated using the reaction-field method, while the van der Waals interactions were cut off using the potential shift Verlet scheme. A 10-fs integration time step was used. The results from these CG simulations were then compared to all-atom simulations of TCP_96_ performed using the CHARMM36m forcefield [49]. We found that Martini 3 forcefield is better at recapitulating the dynamics of the peptide from all-atom simulations, particularly the flexibility of helix at residues 80–96, the putative heparin binding exosite (Fig. S5). Hence, the Martini 3 forcefield was used for CG simulations of TCP_96_ with heparin.

The Martini 3 CG parameters for heparin were adapted from our CG model of glycosaminoglycans (GAGs) developed for the Martini 2.2 forcefield [50]. The heparin strand is made of 20 sugar units (20-mer) comprising 10 repeat disaccharides of 2-O-sulfated iduronic acid-α1,4-N,6-O-sulfated glucosamine (Fig. S6A). A total of 10 copies of TCP_96_ peptides and 10 strands of heparin were placed in a 25 × 25 × 25 nm^3^ box, with a minimum distance of 2 nm between each molecule. The box was solvated with Martini water particles and neutralized with NaCl salt. Energy minimization was performed using the steepest descent method followed by a 100 ns equilibration simulation, whereby positional restraints with a force constant of 1000 kJ mol^−1^ nm^−2^ were applied to the backbone beads of the peptide and all beads of heparin. Three independent 10 μs production simulations were performed using the CG Martini 3 forcefield [46] and GROMACS 2021 [51]. The same simulation protocols as described above were used, with the exception of the integration time step. For these systems, a 5-fs simulation time step was used instead.

### All-atom molecular dynamics simulation of TCP_96_ with heparin and heparan sulfate

The crystal structure of human α-thrombin bound to a heparin fragment (PDB:1XMN) [21] was used as the starting structure to model TCP_96_ bound to heparin and heparan sulfate (HS). The thrombin light chain and residues 1–158 of the thrombin heavy chain were removed, leaving the last 96 residues to form TCP_96_, corresponding to 11 kDa TCP. Since the structure is missing the C-terminal E96 residue, a higher-resolution structure of unliganded human α-thrombin (PDB:3U69) [52] was used to model the residue using Modeller 9.21 [53]. The heparin fragment is made of three repeat disaccharide units of 2-O-sulfated iduronic acid-α1,4-N,6-O-sulfated glucosamine (Fig. S6B). Each disaccharide has 3 sulfate groups, hence the whole fragment contains 9 total sulfate moieties. To model HS with different sulfation levels, the CHARMM-GUI Glycan Reader and Modeller was used [54]. The sulfate groups were gradually removed, first from the 6-O position on glucosamine, followed by the 2-O position on iduronic acid (which is then converted to glucuronic acid), and finally from the N- position on glucosamine (Fig. S6C). This follows the inverse of established HS biosynthesis and modification pathways in the cell [55]. Our modelling thus produced TCP_96_ bound to HS containing 1 to 8 sulfate moieties.

All-atom molecular dynamics (MD) simulations were performed to investigate the effect of sulfation on TCP_96_-heparin/HS binding. The TCP_96_-heparin/HS complex was first solvated with TIP3P water molecules and neutralized with 0.15 M NaCl salt. Energy minimization was then conducted using the steepest descent method and a 125 ps equilibration simulation was run following the standard CHARMM-GUI protocols [56]. For production simulations, three replicates of 1000 ns runs were performed for each system using GROMACS 2021 [51] and the CHARMM36m force field [49]. For the sugar molecules, we used the CHARMM all-atom force field for carbohydrate derivatives [57], which has been shown to reliably reproduce the major pucker conformations of sugar molecules observed in experiments including that of glucosamine, iduronic acid, and glucuronic acid [58]. Temperature and pressure were coupled to the Nosé-Hoover thermostat [59,60] and Parrinello-Rahman barostat [61], respectively, to maintain 310 K and 1 atm. Coulombic interactions were computed using the particle mesh Ewald (PME) method [62], while the Lennard-Jones interactions were truncated at 1.2 nm with a force switch function applied between 1.0 and 1.2 nm. A 2-fs integration time step was used with all covalent bonds involving hydrogen atoms constrained using the LINCS algorithm [63]. Analyses of simulation trajectories were conducted using GROMACS 2021 tools, while visualization was performed using VMD [64].

### Microscale thermophoresis (MST)

MST was performed as described previously [8]. Briefly, rTCP_96_ (10 μM) was labeled by Monolith NT Protein labelling kit RED–NHS (Nano Temper Technologies) according to the manufacturer's protocol. Then, 5 μL of rTCP_96_ (1.5 μM) were mixed with increasing doses of different GAGs (0.15–5000 μg/mL) in 10 mM Tris at pH 7.4. Then, samples were loaded into standard glass capillaries (Monolith NT Capillaries, Nano Temper Technologies) and the MST analysis was performed on NanoTemper Monolith NT.115 apparatus (Nano Temper Technologies). The light-emitting diode and infrared laser were set to 80 %. Results shown are mean values ± SD of six measurements.

### Statistical analysis

All the experiments were performed at least 3 times. The results are presented as the means ± SD or SEM as indicated. The data were analyzed using GraphPad Prism (GraphPad Software, Inc., USA), and *P* ≤ 0.05 was considered significant. *P* values were determined using one-way ANOVA with Dunnett's or Tukey's multiple comparison test.

## CRediT authorship contribution statement

**Ganna Petruk:** Writing – review & editing, Writing – original draft, Visualization, Validation, Supervision, Project administration, Methodology, Investigation, Funding acquisition, Formal analysis, Data curation, Conceptualization. **Jitka Petrlova:** Writing – review & editing, Methodology, Investigation, Formal analysis, Data curation, Conceptualization. **Firdaus Samsudin:** Writing – review & editing, Writing – original draft, Methodology, Investigation, Formal analysis. **Peter J. Bond:** Writing – review & editing, Supervision, Funding acquisition. **Artur Schmidtchen:** Writing – original draft, Supervision, Resources, Funding acquisition, Conceptualization.

## Declaration of competing interest

The authors declare the following financial interests/personal relationships which may be considered as potential competing interests: Artur Schmidtchen reports a relationship with In2cure AB that includes: board membership. Artur Schmidtchen reports a relationship with Xinnate AB that includes: board membership. Ganna Petruk reports a relationship with Xinnate AB that includes: employment. A.S. is a founder of In2cure AB, a parent company of Xinnate AB, companies that are developing therapies based on thrombin-derived peptides and variants. G.P. is employed part-time by Xinnate AB. The other authors, they declare that they have no known competing financial interests or personal relationships that could have appeared to influence the work reported in this paper.

## References

[1] Chen G.Y., Nunez G. (2010). Sterile inflammation: sensing and reacting to damage. Nat. Rev. Immunol..

[2] Murao A., Aziz M., Wang H., Brenner M., Wang P. (2021). Release mechanisms of major DAMPs. Apoptosis.

[3] Pittman K., Kubes P. (2013). Damage-associated molecular patterns control neutrophil recruitment. J. Innate Immun..

[4] Campbell E.J. (1986). Preventive therapy of emphysema. Lessons from the elastase model. Am. Rev. Respir. Dis..

[5] Eming S.A., Krieg T., Davidson J.M. (2007). Inflammation in wound repair: molecular and cellular mechanisms. J. Invest. Dermatol..

[6] Peplow P.V. (2005). Glycosaminoglycan: a candidate to stimulate the repair of chronic wounds. Thromb Haemost.

[7] Papareddy P., Rydengard V., Pasupuleti M., Walse B., Morgelin M., Chalupka A., Malmsten M., Schmidtchen A. (2010). Proteolysis of human thrombin generates novel host defense peptides. PLoS Pathog..

[8] Petrlova J., Hansen F.C., van der Plas M.J.A., Huber R.G., Morgelin M., Malmsten M., Bond P.J., Schmidtchen A. (2017). Aggregation of thrombin-derived C-terminal fragments as a previously undisclosed host defense mechanism. Proc Natl Acad Sci U S A.

[9] Petrlova J., Petruk G., Huber R.G., McBurnie E.W., van der Plas M.J.A., Bond P.J., Puthia M., Schmidtchen A. (2020). Thrombin-derived C-terminal fragments aggregate and scavenge bacteria and their proinflammatory products. J. Biol. Chem..

[10] Yanagishita M., Hascall V.C. (1992). Cell surface heparan sulfate proteoglycans. J. Biol. Chem..

[11] Gallagher J.T., Lyon M., Steward W.P. (1986). Structure and function of heparan sulphate proteoglycans. Biochem. J..

[12] Song Y., Zhang F., Linhardt R.J. (2021). Analysis of the glycosaminoglycan chains of proteoglycans. J. Histochem. Cytochem..

[13] Goodall K.J., Poon I.K., Phipps S., Hulett M.D. (2014). Soluble heparan sulfate fragments generated by heparanase trigger the release of pro-inflammatory cytokines through TLR-4. PLoS One.

[14] Akbarshahi H., Axelsson J.B., Said K., Malmstrom A., Fischer H., Andersson R. (2011). TLR4 dependent heparan sulphate-induced pancreatic inflammatory response is IRF3-mediated. J. Transl. Med..

[15] Collins L.E., Troeberg L. (2019). Heparan sulfate as a regulator of inflammation and immunity. J. Leukoc. Biol..

[16] Kjellen L., Lindahl U. (2018). Specificity of glycosaminoglycan-protein interactions. Curr. Opin. Struct. Biol..

[17] Brower M.S., Walz D.A., Garry K.E., Fenton J.W. (1987). Human neutrophil elastase alters human alpha-thrombin function: limited proteolysis near the gamma-cleavage site results in decreased fibrinogen clotting and platelet-stimulatory activity. Blood.

[18] Tersariol I.L., Pimenta D.C., Chagas J.R., Almeida P.C. (2002). Proteinase activity regulation by glycosaminoglycans. Braz. J. Med. Biol. Res..

[19] Voynow J.A., Zheng S., Kummarapurugu A.B. (2020). Glycosaminoglycans as multifunctional anti-elastase and anti-inflammatory drugs in cystic fibrosis lung disease. Front. Pharmacol..

[20] Casu B. (1990). Heparin structure. Haemostasis.

[21] Carter W.J., Cama E., Huntington J.A. (2005). Crystal structure of thrombin bound to heparin. J. Biol. Chem..

[22] Holdbrook D.A., Singh S., Choong Y.K., Petrlova J., Malmsten M., Bond P.J., Verma N.K., Schmidtchen A., Saravanan R. (2018). Influence of pH on the activity of thrombin-derived antimicrobial peptides. Biochim. Biophys. Acta Biomembr..

[23] Kasetty G., Papareddy P., Kalle M., Rydengard V., Morgelin M., Albiger B., Malmsten M., Schmidtchen A. (2011). Structure-activity studies and therapeutic potential of host defense peptides of human thrombin. Antimicrob. Agents Chemother..

[24] Fransson L.A., Sjoberg I., Havsmark B. (1980). Structural studies on heparan sulphates. Characterization of oligosaccharides; obtained by periodate oxidation and alkaline elimination. Eur. J. Biochem..

[25] Larsen J.B., Hvas A.M. (2021). Thrombin: a pivotal player in hemostasis and beyond. Semin. Thromb. Hemost..

[26] Kalle M., Papareddy P., Kasetty G., Morgelin M., van der Plas M.J., Rydengard V., Malmsten M., Albiger B., Schmidtchen A. (2012). Host defense peptides of thrombin modulate inflammation and coagulation in endotoxin-mediated shock and Pseudomonas aeruginosa sepsis. PLoS One.

[27] Papareddy P., Kalle M., Sorensen O.E., Malmsten M., Morgelin M., Schmidtchen A. (2013). The TFPI-2 derived peptide EDC34 improves outcome of gram-negative sepsis. PLoS Pathog..

[28] Saravanan R., Holdbrook D.A., Petrlova J., Singh S., Berglund N.A., Choong Y.K., Kjellstrom S., Bond P.J., Malmsten M., Schmidtchen A. (2018). Structural basis for endotoxin neutralisation and anti-inflammatory activity of thrombin-derived C-terminal peptides. Nat. Commun..

[29] van der Plas M.J., Bhongir R.K., Kjellstrom S., Siller H., Kasetty G., Morgelin M., Schmidtchen A. (2016). Pseudomonas aeruginosa elastase cleaves a C-terminal peptide from human thrombin that inhibits host inflammatory responses. Nat. Commun..

[30] Frommherz K.J., Faller B., Bieth J.G. (1991). Heparin strongly decreases the rate of inhibition of neutrophil elastase by alpha 1-proteinase inhibitor. J. Biol. Chem..

[31] Spencer J.L., Stone P.J., Nugent M.A. (2006). New insights into the inhibition of human neutrophil elastase by heparin. Biochemistry.

[32] Ferreira A.V., Perelshtein I., Perkas N., Gedanken A., Cunha J., Cavaco-Paulo A. (2017). Detection of human neutrophil elastase (HNE) on wound dressings as marker of inflammation. Appl. Microbiol. Biotechnol..

[33] Hasmann A., Gewessler U., Hulla E., Schneider K.P., Binder B., Francesko A., Tzanov T., Schintler M., Van der Palen J., Guebitz G.M., Wehrschuetz-Sigl E. (2011). Sensor materials for the detection of human neutrophil elastase and cathepsin G activity in wound fluid. Exp. Dermatol..

[34] Engelberg H. (1961). Plasma heparin levels in normal man. Circulation.

[35] Rein C.M., Desai U.R., Church F.C. (2011). Serpin-glycosaminoglycan interactions. Methods Enzymol..

[36] Campbell E.J., Owen C.A. (2007). The sulfate groups of chondroitin sulfate- and heparan sulfate-containing proteoglycans in neutrophil plasma membranes are novel binding sites for human leukocyte elastase and cathepsin G. J. Biol. Chem..

[37] Redini F., Tixier J.M., Petitou M., Choay J., Robert L., Hornebeck W. (1988). Inhibition of leucocyte elastase by heparin and its derivatives. Biochem. J..

[38] Penc S.F., Pomahac B., Winkler T., Dorschner R.A., Eriksson E., Herndon M., Gallo R.L. (1998). Dermatan sulfate released after injury is a potent promoter of fibroblast growth factor-2 function. J. Biol. Chem..

[39] Huang R., Zong C., Venot A., Chiu Y., Zhou D., Boons G.J., Sharp J.S. (2016). De novo sequencing of complex mixtures of heparan sulfate oligosaccharides. Anal. Chem..

[40] Turnbull J.E., Hopwood J.J., Gallagher J.T. (1999). A strategy for rapid sequencing of heparan sulfate and heparin saccharides. Proc Natl Acad Sci U S A.

[41] Gray C.J., Migas L.G., Barran P.E., Pagel K., Seeberger P.H., Eyers C.E., Boons G.J., Pohl N.L.B., Compagnon I., Widmalm G., Flitsch S.L. (2019). Advancing solutions to the carbohydrate sequencing challenge. J. Am. Chem. Soc..

[42] Zsila F., Samsonov S.A., Maszota-Zieleniak M. (2020). Mind your dye: the amyloid sensor thioflavin T interacts with sulfated glycosaminoglycans used to induce cross-beta-sheet motifs. J. Phys. Chem. B.

[43] Petrlova J., Bhattacherjee A., Boomsma W., Wallin S., Lagerstedt J.O., Irback A. (2014). Conformational and aggregation properties of the 1-93 fragment of apolipoprotein A-I. Protein Sci..

[44] Morrisett J.D., David J.S., Pownall H.J., Gotto A.M. (1973). Interaction of an apolipoprotein (apoLP-alanine) with phosphatidylcholine. Biochemistry.

[45] Monticelli L., Kandasamy S.K., Periole X., Larson R.G., Tieleman D.P., Marrink S.J. (2008). The MARTINI coarse-grained force field: extension to proteins. J. Chem. Theor. Comput..

[46] Souza P.C.T., Alessandri R., Barnoud J., Thallmair S., Faustino I., Grunewald F., Patmanidis I., Abdizadeh H., Bruininks B.M.H., Wassenaar T.A., Kroon P.C., Melcr J., Nieto V., Corradi V., Khan H.M., Domanski J., Javanainen M., Martinez-Seara H., Reuter N., Best R.B., Vattulainen I., Monticelli L., Periole X., Tieleman D.P., de Vries A.H., Marrink S.J. (2021). Martini 3: a general purpose force field for coarse-grained molecular dynamics. Nat. Methods.

[47] Bussi G., Donadio D., Parrinello M. (2007). Canonical sampling through velocity rescaling. J. Chem. Phys..

[48] Parrinello M.R., A (1981). Polymorphic transitions in single crystals: a new molecular dynamics method. J. Appl. Phys..

[49] Huang J., MacKerell A.D. (2013). CHARMM36 all-atom additive protein force field: validation based on comparison to NMR data. J. Comput. Chem..

[50] Shivgan A.T., Marzinek J.K., Krah A., Matsudaira P., Verma C.S., Bond P.J. (2024). Coarse-grained model of glycosaminoglycans for biomolecular simulations. J. Chem. Theor. Comput..

[51] Abraham M.J.M., T, Schulz R., Páll S., Smith J.C., Hess B., Lindahl E. (2015). GROMACS: high performance molecular simulations through multi-level parallelism from laptops to supercomputers. SoftwareX.

[52] Figueiredo A.C., Clement C.C., Zakia S., Gingold J., Philipp M., Pereira P.J. (2012). Rational design and characterization of D-Phe-Pro-D-Arg-derived direct thrombin inhibitors. PLoS One.

[53] Sali A., Blundell T.L. (1993). Comparative protein modelling by satisfaction of spatial restraints. J. Mol. Biol..

[54] Park S.J., Lee J., Qi Y., Kern N.R., Lee H.S., Jo S., Joung I., Joo K., Lee J., Im W. (2019). CHARMM-GUI Glycan Modeler for modeling and simulation of carbohydrates and glycoconjugates. Glycobiology.

[55] Multhaupt H.A., Couchman J.R. (2012). Heparan sulfate biosynthesis: methods for investigation of the heparanosome. J. Histochem. Cytochem..

[56] Lee J., Cheng X., Swails J.M., Yeom M.S., Eastman P.K., Lemkul J.A., Wei S., Buckner J., Jeong J.C., Qi Y., Jo S., Pande V.S., Case D.A., Brooks C.L., MacKerell A.D., Klauda J.B., Im W. (2016). CHARMM-GUI input generator for NAMD, GROMACS, AMBER, OpenMM, and CHARMM/OpenMM simulations using the CHARMM36 additive force field. J. Chem. Theor. Comput..

[57] Guvench O., Mallajosyula S.S., Raman E.P., Hatcher E., Vanommeslaeghe K., Foster T.J., Jamison F.W., Mackerell A.D. (2011). CHARMM additive all-atom force field for carbohydrate derivatives and its utility in polysaccharide and carbohydrate-protein modeling. J. Chem. Theor. Comput..

[58] Guvench O., Martin D., Greene M. (2021). Pyranose ring puckering thermodynamics for glycan monosaccharides associated with vertebrate proteins. Int. J. Mol. Sci..

[59] Hoover W.G. (1985). Canonical dynamics: equilibrium phase-space distributions. Phys Rev A Gen Phys.

[60] Nosé S. (1984). A molecular dynamics method for simulations in the canonical ensemble. Mol. Phys..

[61] Parrinello M.R., A (1981). Polymorphic transitions in single crystals: a new molecular dynamics method. J. Appl. Phys..

[62] Essmann U.P., L, Berkowitz M.L. (1995). A smooth particle mesh Ewald method. J. Chem. Phys..

[63] Hess B.B., H, Berendsen H.J.C., Fraaije Johannes G.E. M., Lincs J.G.E.M. (1997). A linear constraint solver for molecular simulations. J. Comput. Chem..

[64] Humphrey W., Dalke A., Schulten K. (1996). VMD: visual molecular dynamics. J. Mol. Graph..

