# Supplementary material for: Thrombin-derived C-terminal peptides bind and form aggregates with sulfated glycosaminoglycans
Source: Heliyon. 2024 Aug 2;10(16):e35703. doi: 10.1016/j.heliyon.2024.e35703 (PMC11369470; doi:10.1016/j.heliyon.2024.e35703)
Supplement: Multimedia component 1 [file mmc1.pdf]

Supplementary Information

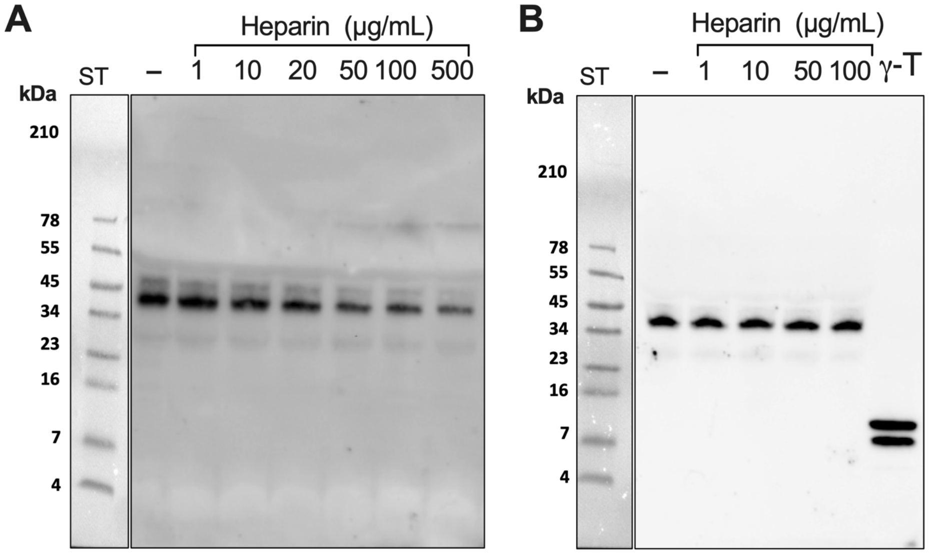

Figure S1

Generation of 11 kDa TCPs in whole blood or serum. (A-B) A representative image of SDS-PAGE, followed by western blotting, using specific antibodies against the C-terminal thrombin epitope VFR17 (n = 3). (A) Whole human blood (A) or human serum (B) in the presence of the indicated concentrations of heparin. γ-thrombin (γ-T) was used as a control. (A) The intensity of the bands at 42 kDa, corresponding to molecular mass of α-thrombin, decreases with increasing concentrations of heparin with no formation of TCPs. (B) The intensity of the bands at 42 kDa, corresponding to molecular mass of α-thrombin, remains similar regardless of the concentration of heparin, with no formation of TCPs.

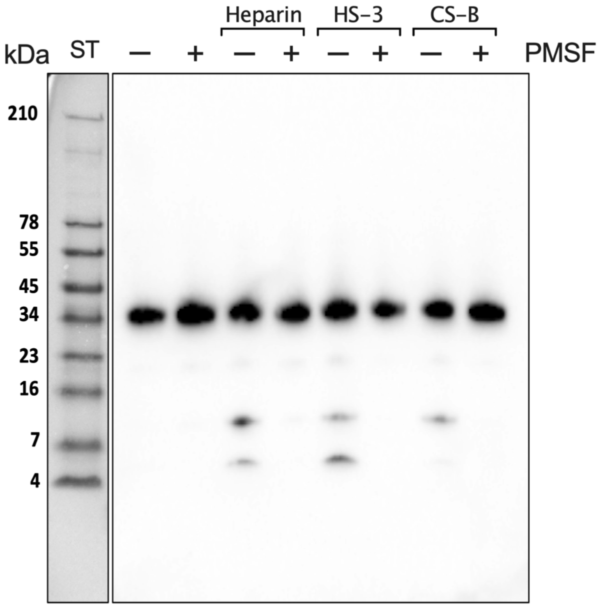

Figure S2

Proteolysis of α-thrombin *in vitro*. A representative image of SDS-PAGE, followed by western blotting, using specific antibodies against the C-terminal thrombin epitope VFR17. α-thrombin in the presence of heparin, HS-3 or CS-B (100 μg/mL) with and without 2 mM protease inhibitors (PMSF) (n = 3). Proteolysis of α-thrombin stimulated by different GAGs is inhibited by PMSF.

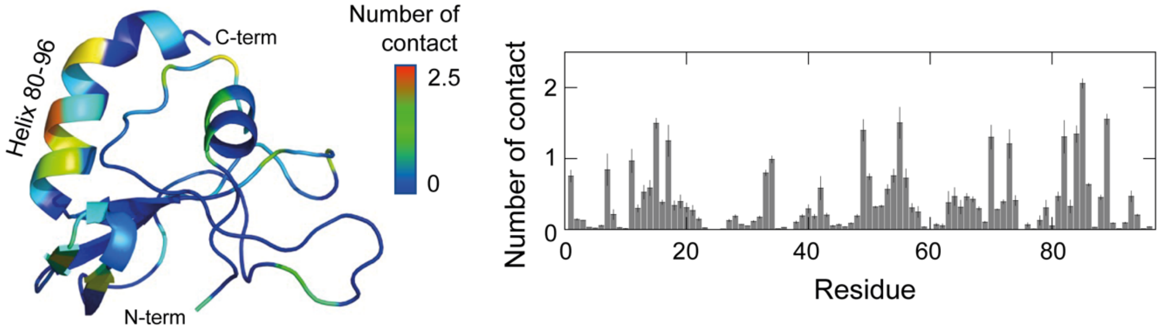

Figure S3

Contact analysis of CG simulations of TCP96 with heparin. The number of contacts made by each residue in TCP96 peptide with heparin from CG simulations were calculated. Average from ten copies of TCP96 and three repeat simulations are shown with the standard deviations depicted as error bars. Distance cut off for contact measurement is 0.6 nm.

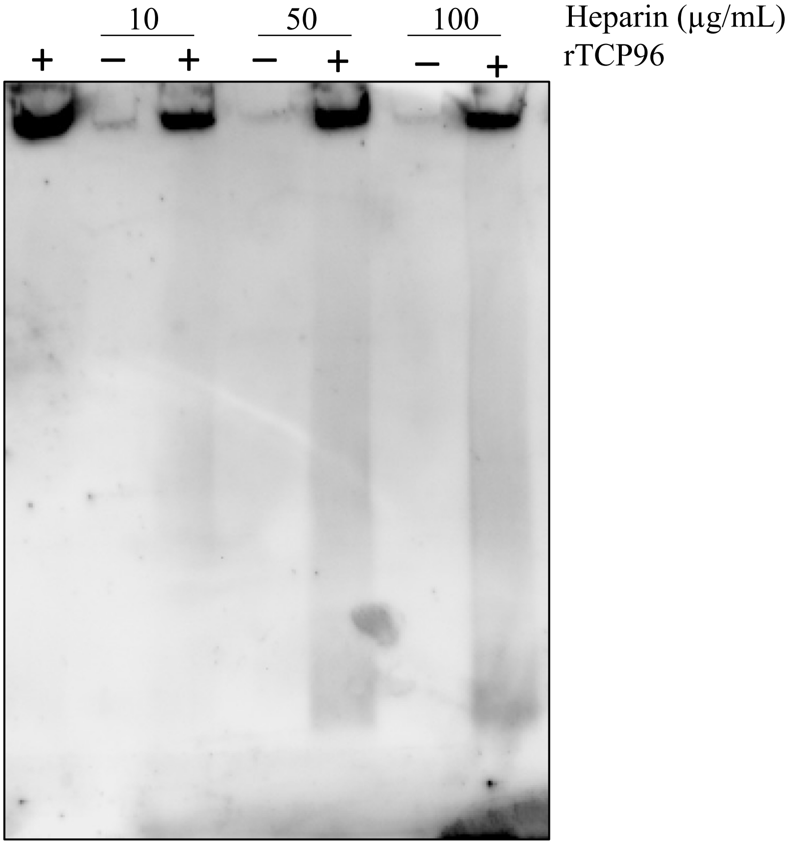

Figure S4

Binding of rTCP96 to heparin. A representative image of BN-PAGE, followed by western blotting, using specific antibodies against the C-terminal thrombin epitope VFR17. Recombinantly produced 11 kDa TCP alone or incubated with different doses of heparin. Buffer containing different doses of heparin was used as a control.

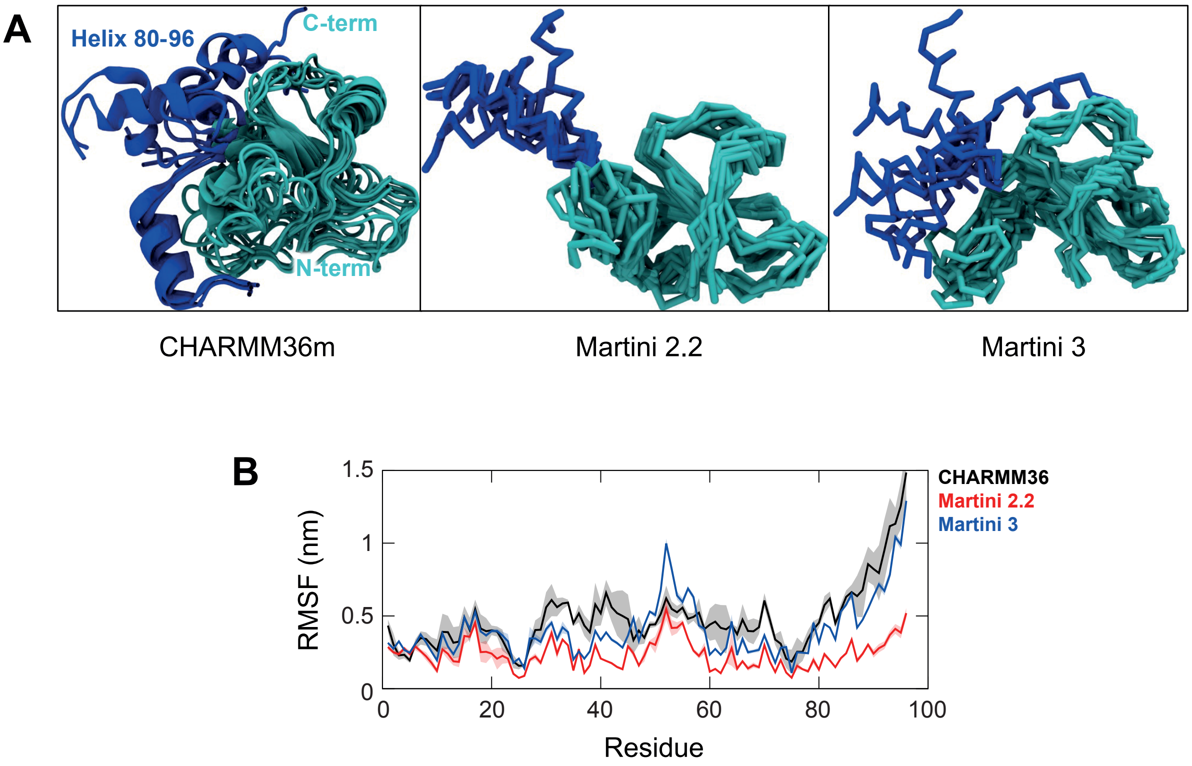

Figure S5

Comparison of atomistic and coarse-grained (CG) simulations of TCP96. (A) Overlaid snapshots of TCP96 peptide taken every 100 ns from a 1000 ns simulation using all-atom CHARMM36m forcefield (left), CG MARTINI 2.2 forcefield (middle) and CG MARTINI 3 forcefield (right). Helix 80-96, which forms the putative heparin binding exosite in thrombin, is shown in blue, and the rest of the peptide is in cyan. (B) Average per-residue root means square fluctuation (RMSF) from three independent repeats. Shaded regions indicate standard deviations between repeats.

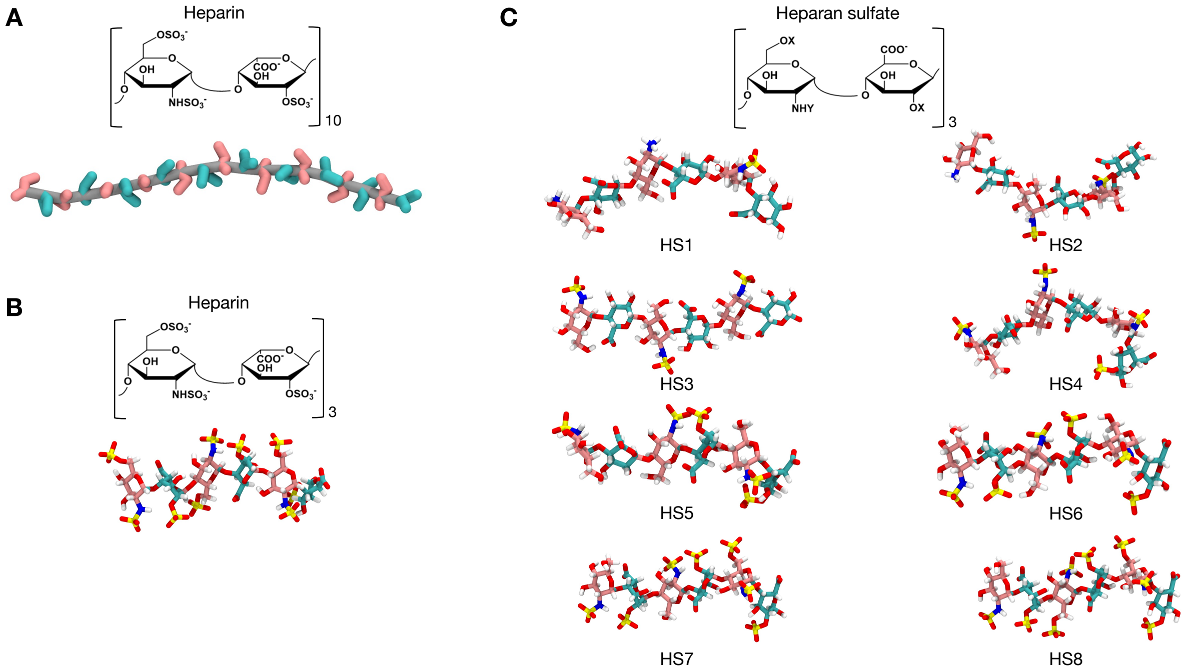

Figure S6

Structures of glycosaminoglycans used in this study. (A) (Top) The chemical structure of heparin used in the CG simulations with TCP_{96}. (Bottom) A CG model of heparin coloured in pink for glucosamine, cyan for iduronic acid, and grey for glycosidic linkages. (B) (Top) The chemical structure of heparin used in the all-atom simulations with TCP_{96}. (Bottom) An all-atom model of heparin coloured in pink for glucosamine and cyan for iduronic acid. The sulfate groups are shown in yellow. (C) (Top) A representative chemical structure of heparan sulfate used in the all-atom simulations with TVP_{96}. X=H or SO_3^- ; Y=$COCH_3$ or SO_3^- . (Bottom) All-atom models of heparan sulfate with varying number of sulfate groups coloured in pink for glucosamine, cyan for iduronic/glucuronic acid, and yellow for sulfate groups.
